# Editorial to the Special Issue “Gulliver in the Country of Lilliput: An Interplay of Noncovalent Interactions (Volume II)”

**DOI:** 10.3390/molecules30142972

**Published:** 2025-07-15

**Authors:** Ilya G. Shenderovich

**Affiliations:** Institute of Organic Chemistry, University of Regensburg, Universitaetstrasse 31, 93053 Regensburg, Germany; ilya.shenderovich@ur.de

Noncovalent interactions are the bridge between the ideal gas abstraction and the real world. In the past, most studies were limited to the analysis of the single strongest interaction in a molecular system under consideration, which was held responsible for the most important structural properties of the system. The current challenge is to surpass this limitation.

The first edition of the Special Issue, “Gulliver in the Country of Lilliput: An Interplay of Noncovalent Interactions”—which has been published as a standalone book—was successful [1]. As such, the overarching goal of this accompanying Special Issue was to gather insights into the interplay of noncovalent interactions within complex molecular systems, including the roles of cooperativity and anti-cooperativity, solvation, reaction fields, steric hindrance, intermolecular dynamics, and numerous other factors affecting molecular conformation, chemical reactivity, and the structure of condensed matter.

The contributions that comprise this Special Issue can be broadly categorized into three main themes: (i) the identification of specific interactions responsible for the observed or predicted structures of heteromolecular systems [2,3,4]; (ii) the role of noncovalent interactions in the behavior of molecules under confinement [5,6,7]; and (iii) the spectral manifestations of such interactions in complex molecular systems [8,9,10,11].

The experimental study of Mihrin et al. [2] reports the structures of the phenol dimer, trimer, and monohydrate embedded in inert neon matrices at 4 K. Specifically, large-amplitude hydrogen-bond librational motions were measured and interpreted in terms of the molecular structures of the corresponding complexes using extensive conformational searches and computational optimizations employing both dispersion-corrected DFT and wavefunction ab initio methodologies. Phenol clusters were found to exhibit, in addition to the energy-dominating O–H···O cooperative hydrogen bonds, C–H···π contacts and competing London dispersion forces between the aromatic rings. In the phenol monohydrate, phenol behaves as the hydrogen-bond donor to water, in contrast to aliphatic alcohols, which act as acceptors [12].

Rosokha et al. [3] investigated the role of halogen bonding in N-chlorosuccinimide (SimCl) reactions with bromide and iodide. X-ray crystallography revealed halogen-exchange products in both cases, exhibiting exceptionally short Br···Cl and I···Cl contacts. DFT computations showed that SimCl·Br^−^ and SimCl·I^−^ complexes dissociate into succinimide anions and interhalogen molecules (ClBr or ClI) without high-energy Cl^+^ intermediates, significantly lowering the N–Cl bond dissociation barrier. Consequently, these rearrangements proceed efficiently even at −73 °C. This work highlights that halogen bonds can be strong enough to facilitate chemical reactions [13].

Zhang and Wang [4] used DFT to explore whether 1,2,5-oxadiazole, 2,1,3-benzoxadiazole, and their S-, Se-, and Te-analogues form complexes with fullerene C_60_ via an N→C dative bond [14]. They found that such a bond can form only when assisted by an N–Ch···C_60_ chalcogen bond (Ch = S, Se, Te), driven by σ-holes on the chalcogen. The interaction energies associated with these chalcogen bonds increase by an order of magnitude from S to Te. However, only for 1,2,5-telluradiazole and 2,1,3-benzotelluradiazole does the combined effect of the N→C dative bond and N–Te···C_60_ chalcogen bond overcome the significant repulsive deformation energy of C_60_, making these structures energetically stable. The structures of the corresponding 1,2,5-oxadiazole and 2,1,3-benzoxadiazole complexes with C_60_ are governed by π···π stacking interactions.

In their review, Buntkowsky et al. [5] highlight exemplary studies focusing on the intriguing phase behavior exhibited by guest molecules confined within the pores of MCM-41, SBA-15 mesoporous silicas, and their functionalized derivatives. By combining advanced solid-state NMR techniques with molecular dynamics simulations, the authors demonstrate that despite the cramped pore environments, the balance of intermolecular forces—whether for small molecules such as water or octanol, or for surfactants like C_10_E_6_ or Triton X-100—frequently drives the formation of ordered structures. Notably, guest diffusion within these mesopores is anisotropic and strongly dependent on the filling level [15]. The authors anticipate that ongoing advances in molecular dynamics will soon enable predictive modeling of these confined structures, which can then be validated through targeted experimental studies.

Gómez-Salazar et al. [6] reported the development of a thiol-functionalized silica adsorbent tailored to remove melanoidin-type compounds from organic wastes produced during ethanol fermentation, alongside a UV–Vis spectroscopic method used to monitor the process. Rapid adsorption kinetics and a high capacity for melanoidin removal arise from the direct interaction of the guest with the thiol functional group, a reaction that is well documented [16], rather than with the pristine silica surface. The adsorbent also exhibits high thermal stability, making it well suited for industrial application and helping to reduce wastewater pollution.

Jabłoński [7] presents a theoretical investigation into the factors that govern the selective encapsulation of small guest molecules within the interior cavity of superphanes. In its simplest form, a superphane consists of two aromatic rings held in a rigid, parallel orientation by six –(CH_2_)_2_– linkers, creating a barrel-like hollow core. Modified superphanes, however, incorporate composite binding chains that not only adjust cavity size but also provide specific noncovalent interaction sites for guest molecules, enhancing the trapping selectivity [17]. Through energy-decomposition analyses, the results obtained demonstrate that these engineered binding sites exert a greater influence on selectivity than cavity volume alone.

Melandri et al. [8] describe a combined experimental and theoretical study of the rotational spectrum of the 1:1 acrolein–MeOH (and MeOD) complex in the gas phase. Analysis of the measured spectroscopic parameters unambiguously defines the complex’s conformation, which agrees with high-level quantum-chemical predictions. A notable feature of methanol spectra is the splitting of rotational lines due to hindered internal rotation of the CH_3_ group. In every known 1:1 complex, the barrier to this methyl rotation decreases [18], and in acrolein–MeOH it falls to about 60% of the monomer value. Importantly, this reduction does not correlate with complexation energy but instead arises from coupling with an additional large-amplitude motion. While theory reproduces the qualitative trend, the calculated barrier remains significantly higher than the experimental value. Resolving this quantitative gap will require more sophisticated theoretical treatments in future studies.

Gómez and Cappelli [9] employ aqueous caffeine solutions to illustrate the advantages of a fully polarizable QM/MM approach [19] for (i) predicting the optimal excitation wavelength for resonance Raman spectroscopy and thereby achieving exceptionally low detection limits for caffeine quantification and (ii) elucidating how hydrogen-bond networks shape caffeine’s spectral fingerprints. While the strongest solute–solvent interaction is the N···H–O hydrogen bond between water and caffeine’s imidazole nitrogen, each of caffeine’s two carbonyl groups can simultaneously coordinate up to two water molecules, substantially increasing the stabilization energy of the first hydration shell. It would be highly informative to quantify the entropic penalty associated with this enhanced stabilization and to assess its impact on caffeine’s solubility in water.

Shenderovich [10] surveyed experimental data on how noncovalent interactions perturb the isotropic hyperfine coupling constant of the nitroxide radical TEMPO in a solution. Because these interaction-induced shifts exceed the experimental measurement uncertainty by two orders of magnitude, TEMPO serves as an exceptionally sensitive probe for quantifying weak interactions in solution. These data were used to identify a practical DFT functional and basis-set combination capable of reproducing the observed effects and to assess the utility of TEMPO for studying competing hydrogen bonds (e.g., with 2,2′-bipyridinium [20]) and strong halogen-bond donors. The work demonstrates that accurately interpreting the measured coupling constant changes in terms of specific complex populations requires evaluating many candidate structures, whose relative abundances cannot always be inferred from simple heuristics. Notably, like a P=O moiety [21], the N–O group in TEMPO can simultaneously form two hydrogen bonds of comparable strength.

Stoumpos et al. [11] compared the intramolecular vibrations of pyridinium in one-dimensional lead halide perovskites and in pyridinium halide salts by infrared absorption and Raman scattering. Vibrational bands were rigorously assigned through DFT calculations aided by symmetry analysis. The modes that involve N–H motions exhibit pronounced frequency shifts depending on whether the cation resides within the perovskite channels or in the salt lattice, whereas changing the halide (Br^−^ or I^−^) produces only marginal effects. By analyzing these environment-sensitive shifts, one can qualitatively gauge crystal-structure disorder or detect nonequivalent pyridinium sites. Thus, pyridinium cations in hybrid organic–inorganic crystals serve as intrinsic spectroscopic probes, reporting on their local lattice environment through changes in the selected vibrational frequencies. Similarly, quasi-elastic neutron scattering studies of the rotational dynamics of organic cations have proven effective for probing structural features in perovskites [22].
